# Home-Based EEG Neurofeedback Intervention for the Management of Chronic Pain

**DOI:** 10.3389/fpain.2022.855493

**Published:** 2022-05-27

**Authors:** Nick Birch, Jon Graham, Christine Ozolins, Kaushalya Kumarasinghe, Faisal Almesfer

**Affiliations:** ^1^East Midlands Spine Ltd., Northampton, United Kingdom; ^2^PhysioFunction Ltd., Northampton, United Kingdom; ^3^Exsurgo Ltd., Auckland, New Zealand

**Keywords:** proof-of-concept trial, chronic pain, neurofeedback training, brain computer interface, EEG, patient-reported outcomes

## Abstract

**Background:**

Chronic pain and associated symptoms often cause significant disability and reduced quality of life (QoL). Neurofeedback (NFB) as part of a Brain Computer Interface can help some patients manage chronic pain by normalising maladaptive brain activity measured with electroencephalography (EEG).

**Objectives:**

This study was designed to assess the efficacy and safety of a novel home-based NFB device for managing chronic pain by modifying specific EEG activity.

**Methods:**

A prospective, single-arm, proof-of-concept study was conducted between June 2020 and March 2021 among adults with chronic pain (registered with ClinicalTrials.gov NCT04418362). Axon EEG NFB systems for home use were provided to each, and 32–48 NFB training sessions were completed by the participants over 8-weeks. The primary outcome was self-reported pain. Assessment of central sensitisation, sleep quality, affective symptoms, change in QoL, adverse events during use and EEG correlations with symptoms were secondary outcomes.

**Results:**

Sixteen participants were enrolled. Eleven reported pain relief following NFB training, eight reporting clinically significant improvements. Central sensitisation symptoms improved by a third (*p* < 0.0001), sleep quality by almost 50% (*p* < 0.001), anxiety reduced by 40% (*p* = 0.015), and QoL improved at final follow-up for 13 participants. The majority (69%) of participants who upregulated relative alpha reported improved pain, and those who downregulated relative hi-beta reported improved pain, reduced anxiety and depression scores. There were no adverse events during the trial.

**Conclusions:**

Home-based NFB training is well-tolerated and may provide relief for sufferers of chronic pain and its associated symptoms.

**Summary:**

*Axon*, a home-based NFB training device, can positively influence pain and associated symptoms in a proportion of people with chronic pain.

## Introduction

Chronic pain is a complex multifactorial disease with a wide-ranging aetiology, characterised by pain that lasts or recurs for more than 3 months (1). It is a leading cause of disability worldwide (2, 3) and was classed as a distinct disease state by the World Health Organisation (4).

20–40% of the population are estimated to experience some form of chronic pain (5–7). There is evidence that the COVID-19 pandemic has had a negative influence on the management of chronic pain for existing sufferers, and has been a causative factor in new cases of chronic pain (7, 8).

There is a close relationship between chronic pain and mood disorders, sleep dysfunction, and reduced quality of life (QoL) (7, 9). Anxiety, depression, and sleep dysfunction, independently and in conjunction, have been implicated in the exacerbation of pain perception, and maintenance of the chronic pain disease state (10, 11). All have been shown to have a significant effect on chronic pain outcomes (12).

Chronic pain can be difficult to treat, and many pharmacological treatment strategies are far from optimal (13, 14). They are frequently associated with unwanted side-effects as well as a risk of dependence, misuse, and the development of tolerance (1). Non-pharmacological treatments including supervised exercise programs, cognitive behavioural therapy (CBT) and acceptance and commitment therapy (ACT) offer benefit without the side effects of drugs (1, 15) but can be difficult to access.

An alternative non-pharmacological treatment method is electroencephalography (EEG) neurofeedback (NFB) in combination with a device running bespoke game-based software using the principles of operant conditioning. This forms a therapeutic brain computer interface (BCI) which enables real time self-regulation of brain activity. NFB can be used to “normalise” maladaptive neural signalling observed in chronic pain (16, 17), often characterised by suppressed activity in the alpha frequency range (8–13Hz) in sensorimotor areas of the cerebral cortex (18), i.e., parts of the so-called “Pain Matrix” (19). This maladaptive neural signalling is often accompanied by heightened activity in the upper levels of the beta frequency range—i.e., hi-beta (20–30Hz) (16).

The concept of the “Pain Matrix” represents the activation of an extended network of cortical and sub-cortical brain areas including the somatosensory, insular, cingulate, and prefrontal cortices, the thalamus, and the brainstem (19–21). NFB is believed to work through many brain regions affected by chronic pain.

NFB has been shown to be a safe and effective tool in the treatment of chronic pain (16). However, historically NFB treatments have been expensive, both in terms of clinical time and equipment costs. In a recent study, researchers used a home-based system to test the efficacy of NFB in spinal cord injury patients with chronic neuropathic pain (*n* = 15) (17). Twelve patients achieved statistically significant reductions in pain, and in eight it was clinically significant (>30% reduction in pain).

The current proof-of-concept trial (NCT04418362) sought to replicate the above study by Vučkovic et al. (17) and expand the selection criteria to cover a broader range of chronic pain patients. From this study, the training feedback was determined as increased alpha, decreased hi-beta and theta (if appropriate). It aimed to test the safety and efficacy of a purpose-built headset and tablet-based application designed to be used independently by patients at home (the *Axon* system). The trial objectives were to assess whether NFB training with the *Axon* system could achieve a clinically significant reduction in chronic pain intensity and associated symptoms, and whether any changes would be reflected in altered EEG activity in the alpha, beta or theta frequencies at post-intervention and follow up.

## Methods

### Research Question

Can the use of a home-based EEG neurofeedback system achieve a reduction in chronic pain intensity and associated symptoms without significant side-effects?

### Study Design

Prospective, open label, single arm proof-of-concept study with purposive sampling approved by the Northwest England NHS Research Ethics Committee and registered with ClinicalTrials.gov (NCT04418362).

### Participants

Participants with either primary or secondary chronic pain (or both) according to ICD-11 definitions (i.e., pain that lasts or recurs for more than 3 months), were recruited through clinics and word of mouth, according to the inclusion and exclusion criteria (Table 1). The participant group was heterogenous, representing the three categories of chronic pain—nociceptive, neuropathic and nociplastic (Table 2). At pre-screening, the majority of patients (75%) verbally reported a typical chronic pain level of ≥7 using a scale of 1–10 (10 being the worst pain). Recruitment was successful after completing pre-screening for NFB suitability and fully informed consent according to the Declaration of Helsinki (22). All pre-screening and consent procedures were conducted remotely between June 2020 and December 2020 because of UK COVID-19 restrictions.

**Table 1:** Axon brain train trial inclusion and exclusion criteria.

Inclusion criteria	Chronic pain from any neurological or musculoskeletal cause, including, but not limited to:
	Post-herpetic (or post-shingles) neuralgia, complex regional pain syndrome (CRPS), lower back pain, neck pain, major joint pain, spinal cord injury associated pain, phantom limb pain, brachial plexus injury related pain, traumatic peripheral nerve injury pain and post-cancer treatment pain
	Stable medication and pain management during the intervention period with no anticipated changes in treatment
	Head circumference range 560–595 mm
	Ability of the participant to effectively position and remove headset and operate the tablet PC during training sessions
Exclusion criteria	Dreadlocks, braids, beads, or hairstyle/hair covering that could not be removed for training
	Known or suspected pregnancy
	Current diagnosis of, or currently undergoing treatment for: cancer, systemic infection, severe cardiovascular/respiratory comorbidity
	Implanted electronic neuromodulation device
	Implanted pacemaker or loop recorder

**Table 2:** Participant characteristics.

	**Female**	**Male**
Participants	12	4
Mean age (years)	49.5	52.4
Age range (years)	25–68	29–62
**Diagnosis:**		
Spinal pain	5	2
Peripheral arthritis	2	1
Fibromyalgia	1	1
Neuropathic pain	3	0
Ehlers danlos syndrome	1	0

### Procedure

#### Pre-intervention Assessments

The pre-intervention assessments were: Visual Numeric Scale (VNS) for Pain (23, 24), Central Sensitisation Inventory Part A (CSI-A) (25, 26), Pittsburgh Sleep Quality Index (PSQI) (27), Hospital Anxiety and Depression Scale (HADS) (28, 29), EuroQuol 5 domain, 5 level, QoL Instrument (EQ-5D 5L) (30–32).

The VNS is an 11-point measure of pain intensity at a single point in time. 0 represents no pain and 10 the worst pain imaginable (23, 24). The VNS has been validated against the Visual Analogue Scale (VAS) and was found to be as successful in measuring the underlying pain variable. It is easier to administer and code than the VAS and is sensitive to changes in pain. The CSI-A is used to determine the severity of central sensitisation pain (CSP) (25, 26). It comprises 25 self-reported items recording somatic and emotional symptoms associated with CSP. Each item is graded on a 5-point Likert scale (0: never, 1: rarely, 2: sometimes, 3: often, 4: always). Possible outcome scores range from 0–100: 0–29 indicates subclinical CSP; 30–39 mild CSP; 40–49 moderate CSP; 50–59 severe CSP and 60–100 extreme CSP. The PSQI is a questionnaire that assesses sleep quality (27). There are 19 individual items, that create 7 equally weighted components that are summed to form one global score. The lower the score, the better the sleep quality. The HADS is a simple questionnaire assessing both state anxiety and state depression (scores from 0 to 21 for each, with a lower score indicating a better state) (28, 29). The responses allow categorisation into “non-cases” (scores of <8), “borderline cases” (scores of between 8 and 10) and “cases” (scores of >10). EQ5D-5L is a valid and reliable measure of health-related quality of life consisting of two parts (30–32). Questions related to pain, function and emotional wellbeing in five domains with five possible responses to each for the first. Profiles are constructed that provide an insight into the unique health states of the respondents at the time of completion. A visual analogue “thermometer” of QoL (with a score of between 0 and 100, the higher the score indicating better QoL) forms the second part.

#### Equipment

*Axon* kits, consisting of a purpose-built EEG headset and charger, saline solution, chin strap, tablet PC, stand and charger, and an instruction manual were delivered to the participants by courier.

#### Neurofeedback Training Procedure

Participants were trained remotely *via* secure video link until competency was achieved. During the training they were taught headset fitment, orientation, care, and usage as well as training position and EEG artefact minimisation.

Sessions started with a rating of their current pain and mood and the previous night's sleep, followed by a 2-minute resting state EEG baseline recording (eyes open) looking at a fixation cross on the screen, followed by a 2-minute resting state EEG baseline recording (eyes closed). During baseline recording, relative alpha, theta and hi-beta thresholds were determined. Baseline (eyes open) data was used to calculate the relative alpha threshold for the session, equating to 10% above the average relative alpha. Competency was confirmed when participants could complete a full training session without assistance from the supervising clinician.

During each subsequent NFB training session, once the baseline threshold for the alpha power band had been established, the participants selected their training preference from the following options:

- *Jigsaw Game—*assembling a sequence of jigsaws.
- *Balloon Game—*making a balloon ascend.
- *Bars game—*raising the level of a bar representing alpha activity in a graphic display of the fluctuating alpha, hi-beta, and theta power bands (17).

**Figure 1:**
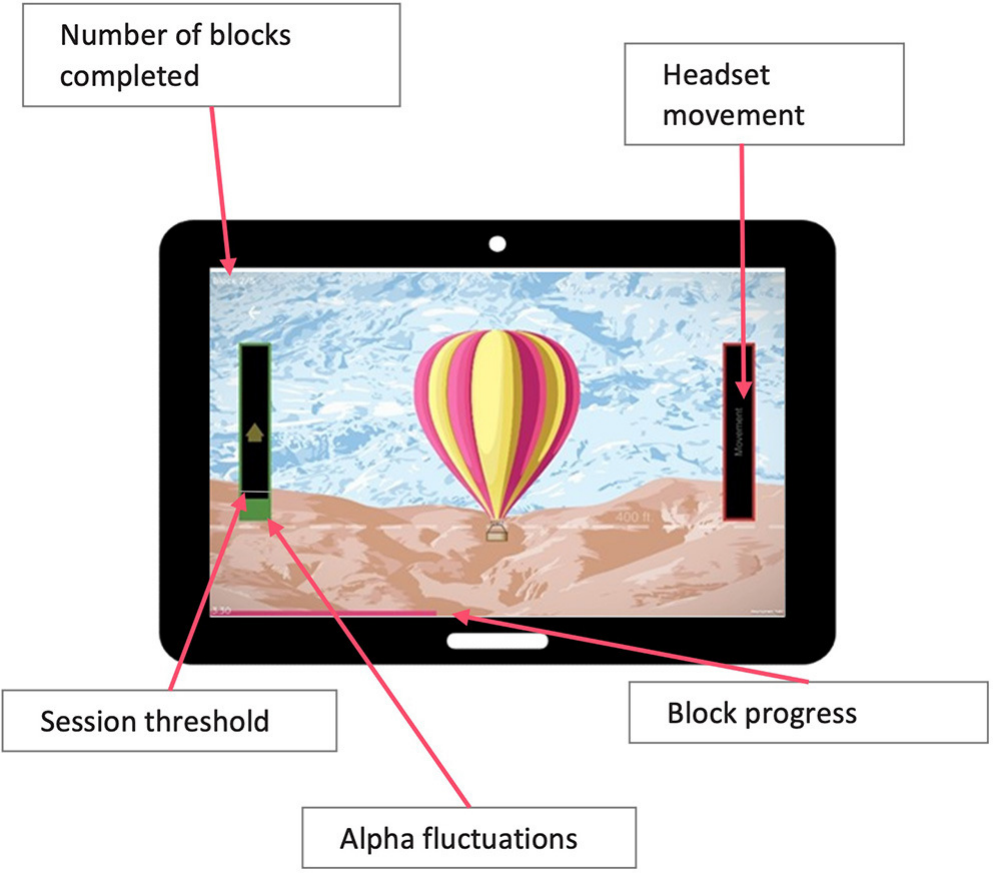
Participants view during training sessions showing representation of their relative alpha threshold on a column to the left of the chosen game.

During training they were instructed to maintain their relaxed seated position, keep movement to a minimum, and “let the game progress.” This way they were able to learn to relax, calm their brain activity, and make the unconscious and conscious connection between relaxation and altered brain activity. No EEG recordings were displayed to patients. Instead, they viewed a gamified representation of their relative alpha threshold on a column to the left of the chosen game (Figure 1). The movement/progression of the game was controlled by the real-time relative alpha power. If the real-time relative alpha power was 10% greater than the 2-minute eyes-open baseline relative alpha power, the game would proceed—i.e., a jigsaw puzzle would assemble itself, a balloon would ascend into the sky, or a set of three bars would turn from pink to green. In addition, the column on the participants screen turned green. When the participant stayed over threshold for 750 ms, they heard an audio tone. Thus, they were rewarded visually for upregulating the alpha band and rewarded with an audio tone for maintaining it, encouraging sustained neural firing within the target range.

To address the potential issue of distraction and enjoyment rather than NFB producing the positive effects on pain, the NFB “games” were of very low stimulus and monotonous. They were designed only to be a way of interacting with subconscious processes of the brain, so enjoyment of the game was not measured. The only driver of game interaction was to make the person feel relaxed, allowing them to self-regulate their alpha band and this process was explained to the patients during the training sessions. As the most important aspect of NFB training is that participants find the positive enforcer that most resonates with them, a choice of different games with differing levels of movement was offered.

Participants performed six 5-minute training blocks, separated by 1-minute rest periods, followed by two post-session baselines (eyes open and eyes closed). At the end of the session, data was uploaded to a secure cloud server for processing in New Zealand.

#### Intervention

Once trained, the participants self-administered 4–6 training sessions a week, aiming to complete 32–48 sessions over a period of 8 weeks. Technical support *via* email, telephone and video link was available throughout the training period. Uploaded EEG data was monitored in New Zealand for electrode impedance, movement artefacts, and any data uploading issues. Problems were resolved via video support calls.

#### Clinical Oversight

Device compliance reviews and possible side-effect assessments were conducted by the clinical investigators during the training period as needed.

#### Post-intervention

At the end of training, participants repeated the primary and secondary outcome questionnaires, a usability questionnaire, and a research participation survey.

Once the intervention was complete, participants were offered continued access to the *Axon* system on compassionate grounds, as agreed with the NHS Ethics Committee.

#### Follow-Up

Follow up assessments were carried out at 4 and 12 weeks after the end of training when the primary and secondary outcome questionnaires were again completed.

### Data Analysis

#### Patient Reported Outcomes

Data collected from the five questionnaires were scored according to each test's criteria. MATLAB (R2021a—The Mathworks, Natick, MA) and Excel for Mac (v 16.53 2021 Microsoft Corp) were used for descriptive and inferential statistical analysis. Statistical significance was tested using one-way ANOVA, the Tukey honestly significant difference (HSD) for post-hoc comparisons between pre- and post-intervention and follow-ups. Clinically significant change in pain was defined as >30% improvement above the VNS baseline (17). Linear least-squares regression to estimate the regulation of alpha and hi-beta, and the Chi-square test for assessing the correlation between changes of EEG and outcome measures were performed using the Python SciPy package (version 1.7.0; R2021) (33).

#### EEG Processing and Analysis

EEG signals were sampled from multiple electrodes located on the scalp, above the somatosensory and prefrontal cortices and transmitted to the *Axon* app via Bluetooth LE. Raw EEG signals from each electrode were captured at 250 Hz and bandpass filtered using multiple Infinity Impulse Response (IIR) filters. The frequency bands of interest were, theta (4–8 Hz), alpha (8–13 Hz), beta (13–30 Hz) and hi-beta (20–30 Hz). Band-pass filtered data were examined for artefact signals, which may be a result of eye blinks, jaw clench or electrode motion. Artefact affected signals were corrected by an estimation algorithm, which estimates the EEG signal, and subsequently discards the contaminated portion.

The band pass filtered data for each frequency band of interest was squared and averaged over a 2 second window. The output of this step was the absolute power of each frequency band.


$$\begin{array}{l} P_{\in }\;(F)=\;\frac{\sum_{i=1}^{n}x^{2}(i)}{n} \end{array}$$

where: *i*=1…n; x = filtered EEG signal; n=window size of 500 samples (2 s of data at 250 samples/s); P_ϵ_ (*F*) = absolute power of specific frequency (*F*).

Relative power of a particular EEG frequency band (*F*) was calculated by finding the ratio between absolute power and absolute broadband power of the concerned band, which was defined as the sum of the absolute powers of the theta, alpha and beta bands.


$$\begin{array}{l} P_{re}\left(F\right)=\frac{P_{\in }\left(F\right)}{P_{\in }\left(\theta \right)+P_{\in }\left(\alpha \right)+P_{\in }\left(\beta \right)} \end{array}$$

where: P_re_(*F*) = relative power of specific frequency (*F*); *P*_ϵ_(*F*) = absolute power of specific frequency (F); *P*_ϵ_(θ) = absolute power of theta band; *P*_ϵ_(α) = absolute power of alpha band; *P*_ϵ_(β) = absolute power of beta band.

Relative alpha, hi-beta and theta were calculated and uploaded to a secure cloud server after each session.

To assess whether the intervention had a significant effect on the targeted alpha band oscillations, a paired two-tailed Student's *t*-test comparing pre- (first five training sessions) and post- (last five training sessions) alpha power (pre-training baseline, eyes-opened) was performed. This method provided a larger, more realistic data set, as alpha activity is a spontaneous but complex rhythm associated with several cognitive states and processes (34).

Linear least-squares regression (33) was performed to estimate the up- or down-regulation of relative alpha, theta and hi-beta power during the intervention. The Pearson correlation coefficient of the linear regression (r) was used to estimate the direction of the trend where r ≥0.2 was considered upregulation and *r* ≤ −0.2 was considered down-regulation of relative alpha, theta or hi-beta power. The *p*-value of the linear regression (whose null hypothesis is that the slope of the linear fit is 0) was used to determine the statistical significance of the estimated EEG up—or down-regulation. The calculated EEG trends together with their statistical significance were used to explore correlations between upregulation of relative alpha and reductions in pain, and down-regulation of hi-beta and reductions in anxiety and depression symptoms.

To assess the correlations of EEG changes after neuromodulation with all measured outcomes, such as the correlation between pre-to-post change in relative alpha and VNS, the Chi-square test of independence of variables using the Python SciPy package was performed (33). The null hypothesis (H0) of the Chi-square test is no correlation between the change in the relative EEG power and outcome measure. The alternative hypothesis (H1) is there is a correlation between the change in the relative EEG power and outcome measures.

## Results

### Participants

**Figure 2:**
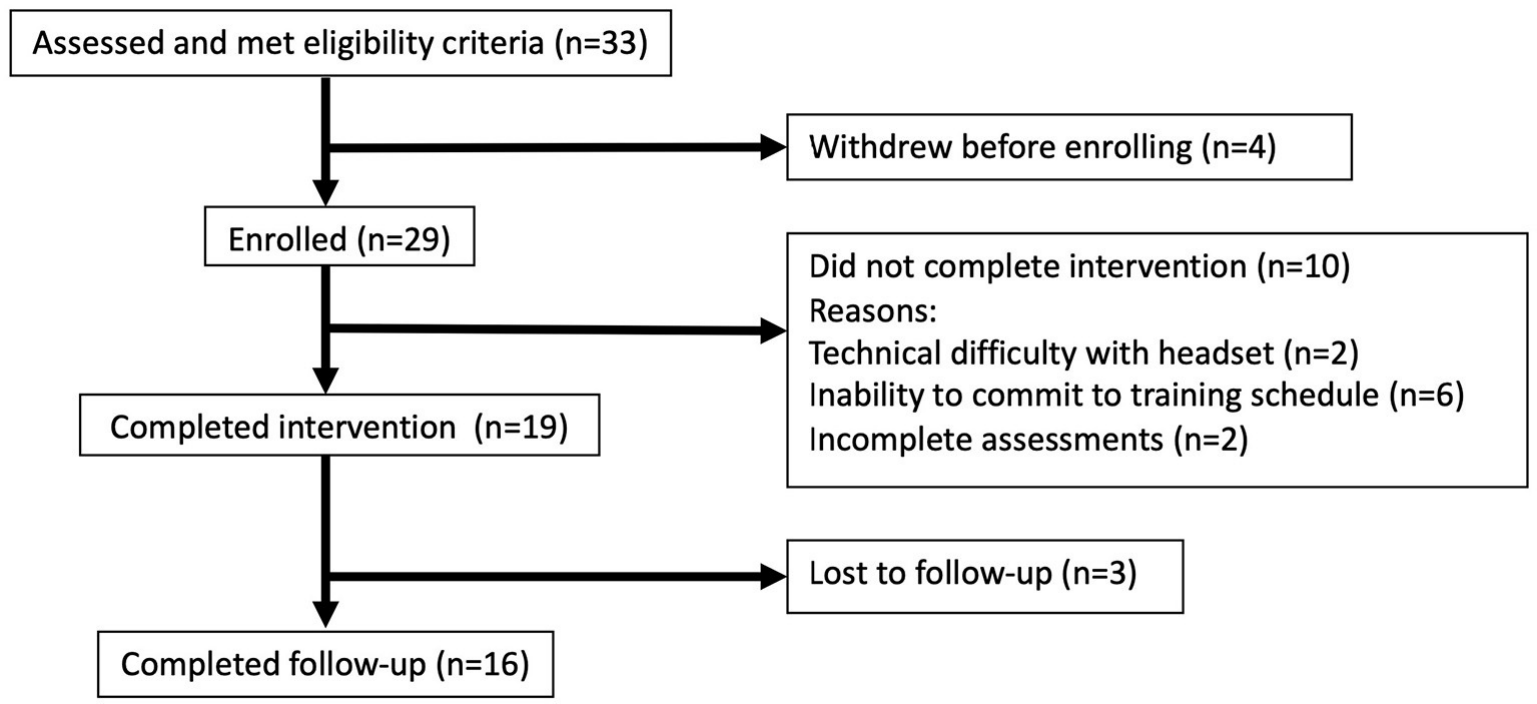
Participant consort diagram showing the process of selection, training and follow-up.

Thirty-three participants aged 25–68 were recruited. Twenty-nine were enrolled, 19 completed the intervention and three were lost to follow up (Figure 2; Table 2).

### Missing Data

There was no missing data during the period of the study, except for one participant who was unable to complete the 12-week follow-up questionnaires. Missing values for this participant were therefore imputed using the group means at 12 weeks.

### Training Sessions

The mean number of sessions completed at the end of the formal training period was 41.7 (range 33–58) and the mean time of training was 32.4 hours (range 23.7–41.6 h). Seven participants completed sessions after the formal end of training (mean 12.0, range 3–35; mean 6.3 h, range 0.75–23.7 h) and the mean number of training sessions when this data was included recorded was 49.0.

### Visual Numeric Scale for Pain

**Figure 3:**
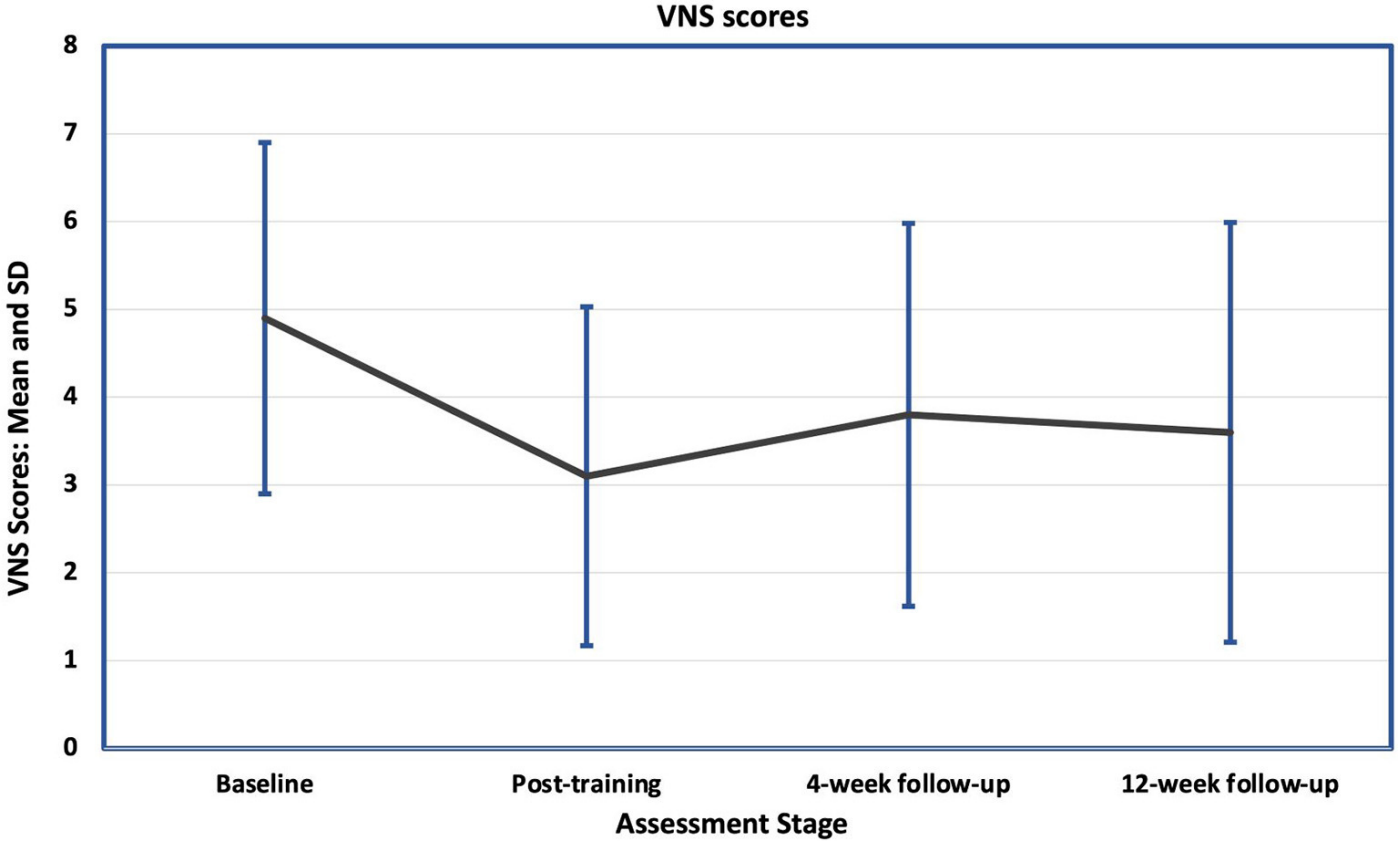
Mean and standard deviations (SD) of the baseline and follow-up Visual Numerical Scale for pain scores (VNS). There was a sustained fall in the mean scores at all follow-up stages, but with large standard deviations, the difference between them and the baseline mean was not significant.

After completing training, 11 participants (69%) reported an improvement in pain, of whom eight achieved a clinically significant improvement (>30% improvement). Two were worse (both reported a single point deterioration) and three reported no change. Mean Visual Numeric Scale (VNS) (Figure 3) improved from 4.9 (SD 2.0) at baseline to 3.1 (SD 1.9) after training (Table 3), a decrease of 37% which was not statistically significant (*p* = 0.104). The mean pain scores at the 4-and 12-weeks follow-ups were 3.75 and 3.5, equating to decreases of 23% and 29% from the pre-intervention scores, which were not statistically significant (*p* = 0.448 and *p* = 0.272 respectively). At 4-weeks follow-up, seven participants (44%) reported a clinically significant improvement and at 12-weeks this rose to eight (50%).

**Table 3:** Summary of results for all 16 participants showing the efficacy of using a novel home-based NFB device for managing chronic pain and its associated symptoms.

**PROM**		**Pre-training**	**Post-training**	**4-week follow-up**	**12-week follow-up**
VNS pain	Mean	4.88	3.13	3.75	3.5
	Range	1–8	0–7	0–7	0–8
	SD	2.0	1.93	2.18	2.39
	*p* (95% CI)		*p* = 0.104 (−0.24–3.74)	*p* = 0.448 (−0.87–3.12)	*p* = 0.272 (−0.62–3.37)
CSI-A	Mean	57.0	37.56	36.31	35.88
	Range	36–85	19–59	18–56	9–54
	SD	11.18	9.76	11.0	11.72
	*p* (95% CI)		*p* < 0.0001 (9.18–29.59)	*p* < 0.0001 (10.41–30.84)	*p* < 0.0001 (10.85–31.28)
PSQI	Mean	11.88	5.44	5.75	6.31
	Range	3–18	1–9	3–11	2–12
	SD	3.88	2.48	2.65	3.36
	*p* (95% CI)		*p* < 0.0001 (3.50–9.37)	*p* < 0.0001 (3.19–9.06)	*p* < 0.0001 (2.63–8.50)
HADS-A	Mean	9.31	6.63	5.63	5.25
	Range	1–19	1–12	0–13	1–10
	SD	4.47	3.30	3.70	3.13
	*p* (95% CI)		*p* = 0.178 (−0.76–6.13)	*p* = 0.031 (0.24–7.13)	*p* = 0.015 (0.62–7.51)
HADS-D	Mean	6.88	3.63	3.81	4.19
	Range	1–18	0–9	0–11	0–11
	SD	4.88	2.68	2.97	2.86
	*p* (95% CI)		*p* = 0.49 (0.01–6.49)	*p* = 0.070 (−0.17–6.30)	*p* = 0.14 (−0.55–5.93)
EQ5D-VAS	Mean	58.2	74.2	73.0	75.94
	Range	34–86	50–90	41–91	45–95
	SD	17.4	11.2	15.2	11.92
	*p* (95% CI)		*p* = 0.01 (−29.24–2.76)	*p* = 0.02 (−28.05–1.58)	*p* = 0.004 (−30.99–4.51)

*Primary and secondary outcome measures are shown with mean, range and standard deviation (SD) for each assessment time point. Statistical significance (p < 0.05) was tested using one-way ANOVA with the Tukey honestly significant difference (HSD) for post-hoc comparisons between pre- and post-intervention and follow-ups.*

*CI, confidence interval; CSI-A, Central Sensitisation Inventory Part A; EQ-5D VAS, EuroQuol 5 domain visual analogue score for quality of life; HADS-A, Hospital Anxiety and Depression Scale—anxiety; HADS-D, Hospital Anxiety and Depression Scale—depression; PROM, patient-reported outcome measure; PSQI, Pittsburgh Sleep Quality Index; VNS, visual numeric scale for pain*.

### Central Sensitisation Inventory

**Figure 4:**
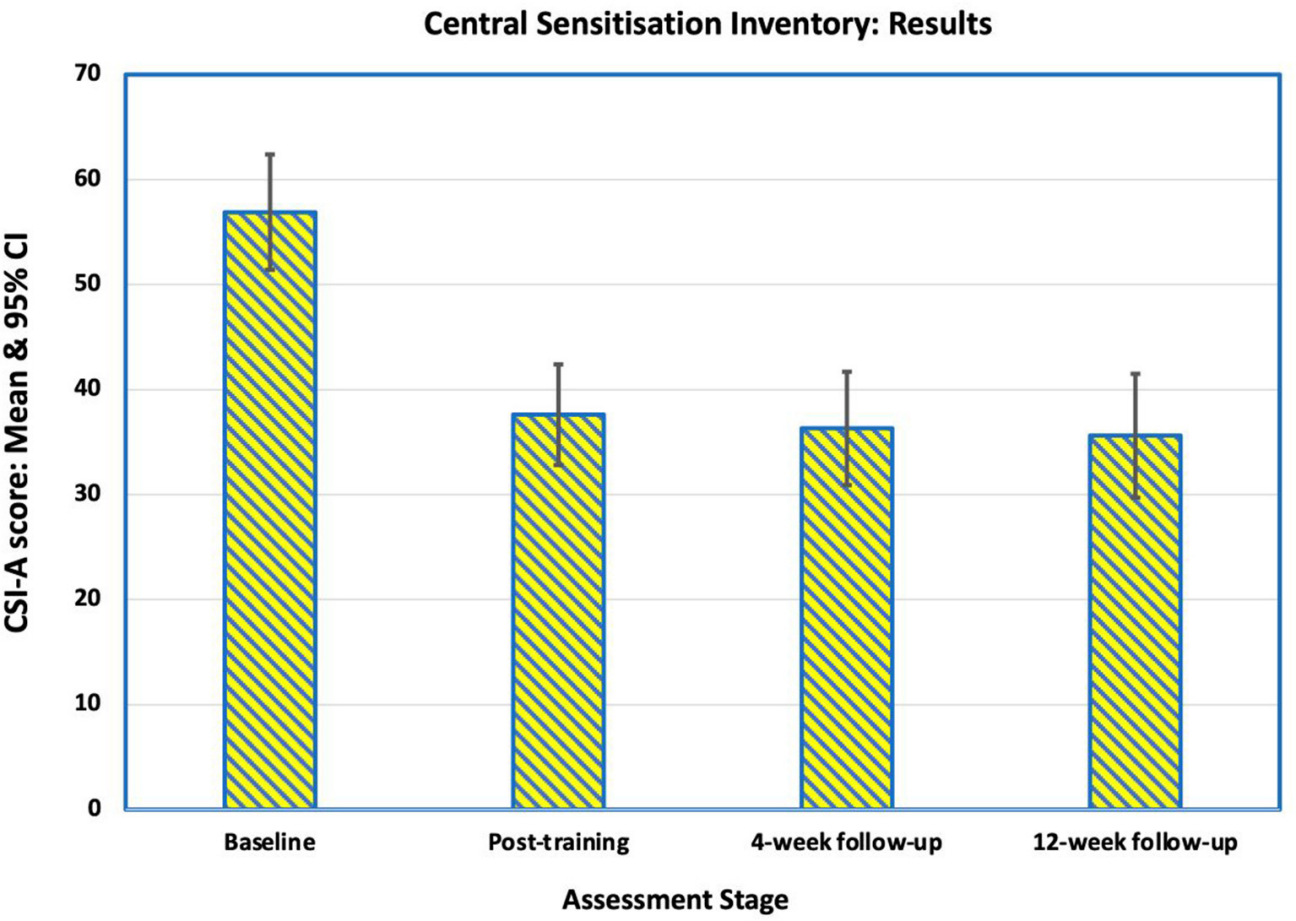
Mean scores on the Central Sensitization Inventory Part A (CSI-A) for the 16 participants at baseline, immediately after training, at 4-week follow-up and at 12-week follow-up. The reduction in mean score indicates an improvement in self-reported symptoms of central sensitization pain.

**Figure 5:**
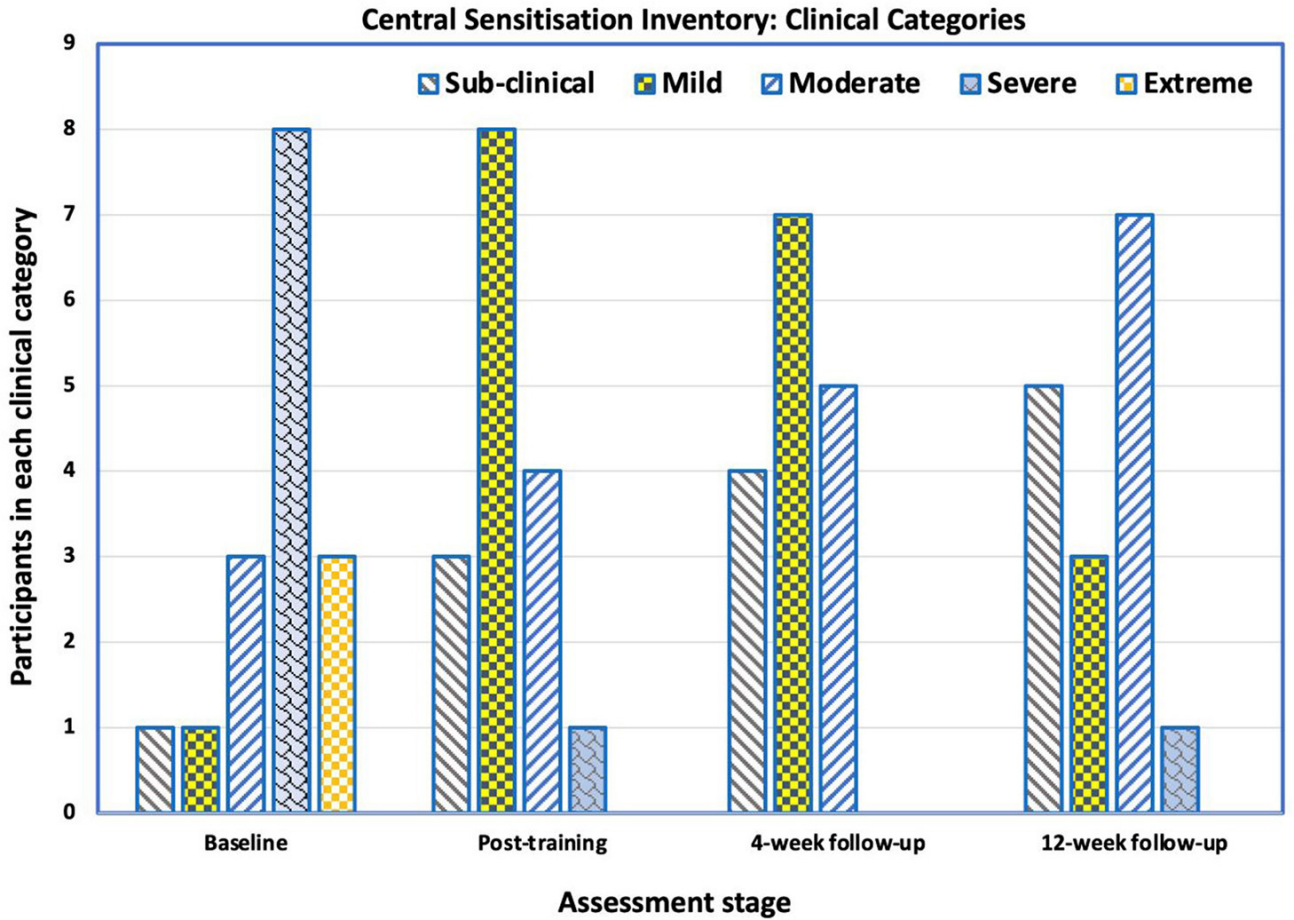
Number of participants in each category (sub-clinical, mild, moderate, severe and extreme) of the Central Sensitization Inventory Part A (CSI-A) across the testing points—baseline, immediately after training, at 4-week follow-up and at 12-week follow-up. There was a shift from 1 out of 16 participants been categorized as having sub-clinical central sensitization pain (CSP) at baseline to 5 participants at the 12-week follow-up. At baseline, 3 of the 16 participants were categorized as having extreme CSP. At the 12-week follow-up, no participants were categorized as having extreme CSP.

Before training, 15 (94%) participants had moderate-to-high levels of central sensitisation (CSI-A score >39), and one (6%) was sub-clinically or mildly affected, which was reflected in the mean Central Sensitisation Inventory (CSI) score of 57 (SD 11.2). There was an improvement in both the mean CSI scores and the number of participants in the sub-clinical/mild groups at all three post-training assessment points (Figures 4, 5). Immediately after training, the mean CSI score fell by 34% to 37.6 (SD 9.8), a level that was maintained at the 4-weeks (mean 36.3, SD 11.0) and 12-weeks follow-ups (mean 35.9, SD 11.7). All three post-intervention CSI means were significantly different from the pre-intervention scores (*p* = 0.001, *p* < 0.0001, *p* < 0.0001 respectively).

### Pittsburgh Sleep Quality Index

**Figure 6:**
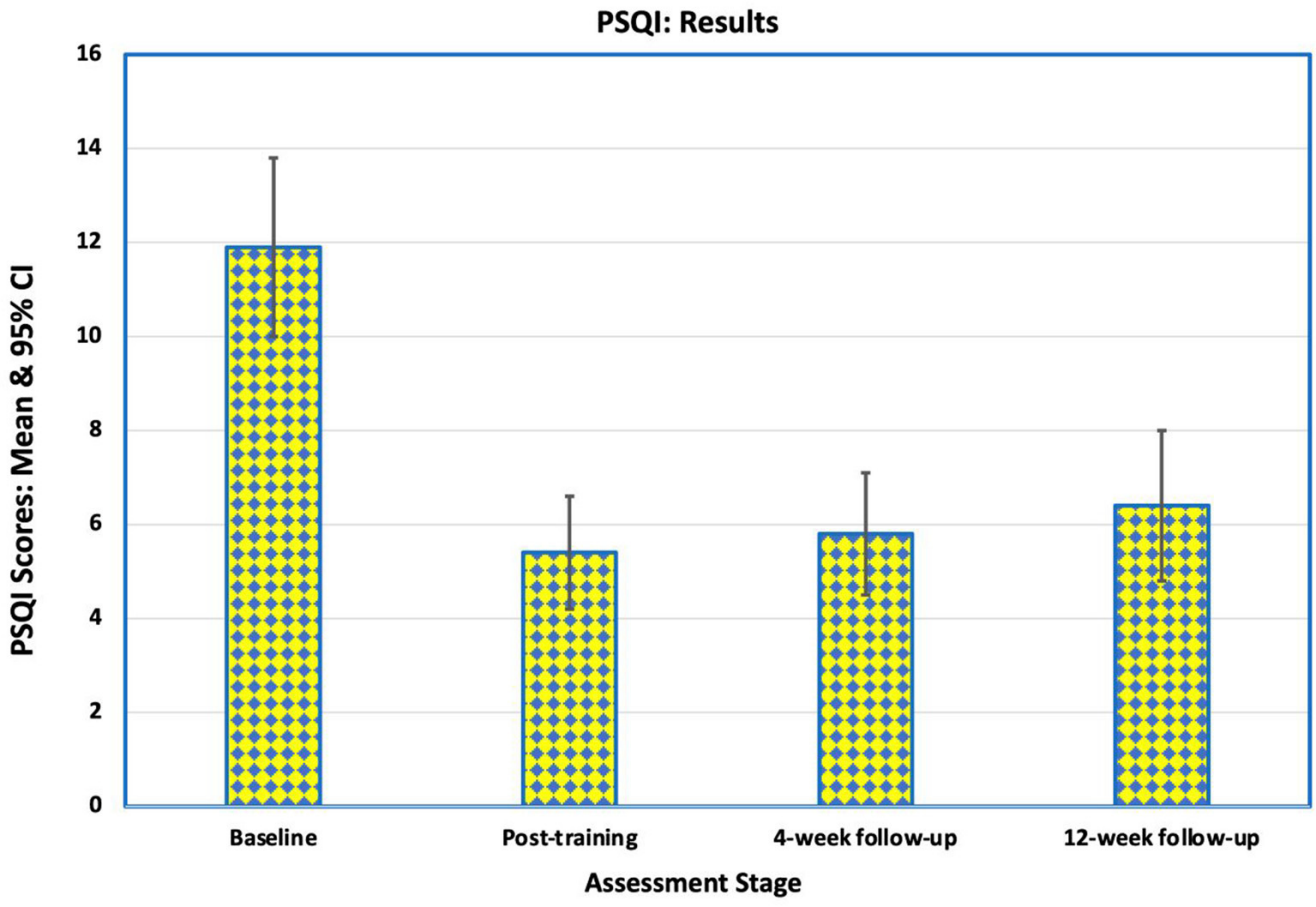
Mean scores on Pittsburgh Sleep Quality Index (PSQI) for the 16 participants at baseline, immediately after training, at 4-week follow-up and at 12-week follow-up. These scores indicate an improvement in the quality of sleep during the study and over the follow-up period. Of the 8 participants who had poor sleep before training and good sleep at the end of training, 7 continued to report good sleep 12 weeks after the intervention.

Before NFB training, 15 of the 16 participants (94%) had poor sleep quality [Pittsburgh Sleep Quality Index (PSQI) > 5]. The mean PSQI was 11.9 (SD 3.9) with a range from 3 to 18. Following training, the PSQI scores fell to a mean of 5.4 (SD 2.5). Nine of the participants reported “good sleep” (PSQI < 6), but seven continued to have poor sleep of whom one had had marginal deterioration in sleep quality. The improvement in sleep quality was maintained for the first 4 weeks after training (mean 5.8, SD 2.6), when 10 participants reported “good sleep” (Figure 6), but there was minor deterioration by 12 weeks post-intervention (mean 6.3, SD 3.4). Improvement in mean PSQI scores at all assessment points after training was statistically significant (*p* < 0.01).

Of the eight participants who had poor sleep before training and good sleep at the end of training, seven continued to report good sleep 12 weeks after the intervention. Three participants reported slightly worse sleep (1 or 2 points higher) at the final follow-up, one of whom had reported good sleep and two of whom had reported poor sleep before training started.

### Hospital Anxiety and Depression Scale

#### Anxiety

**Figure 7:**
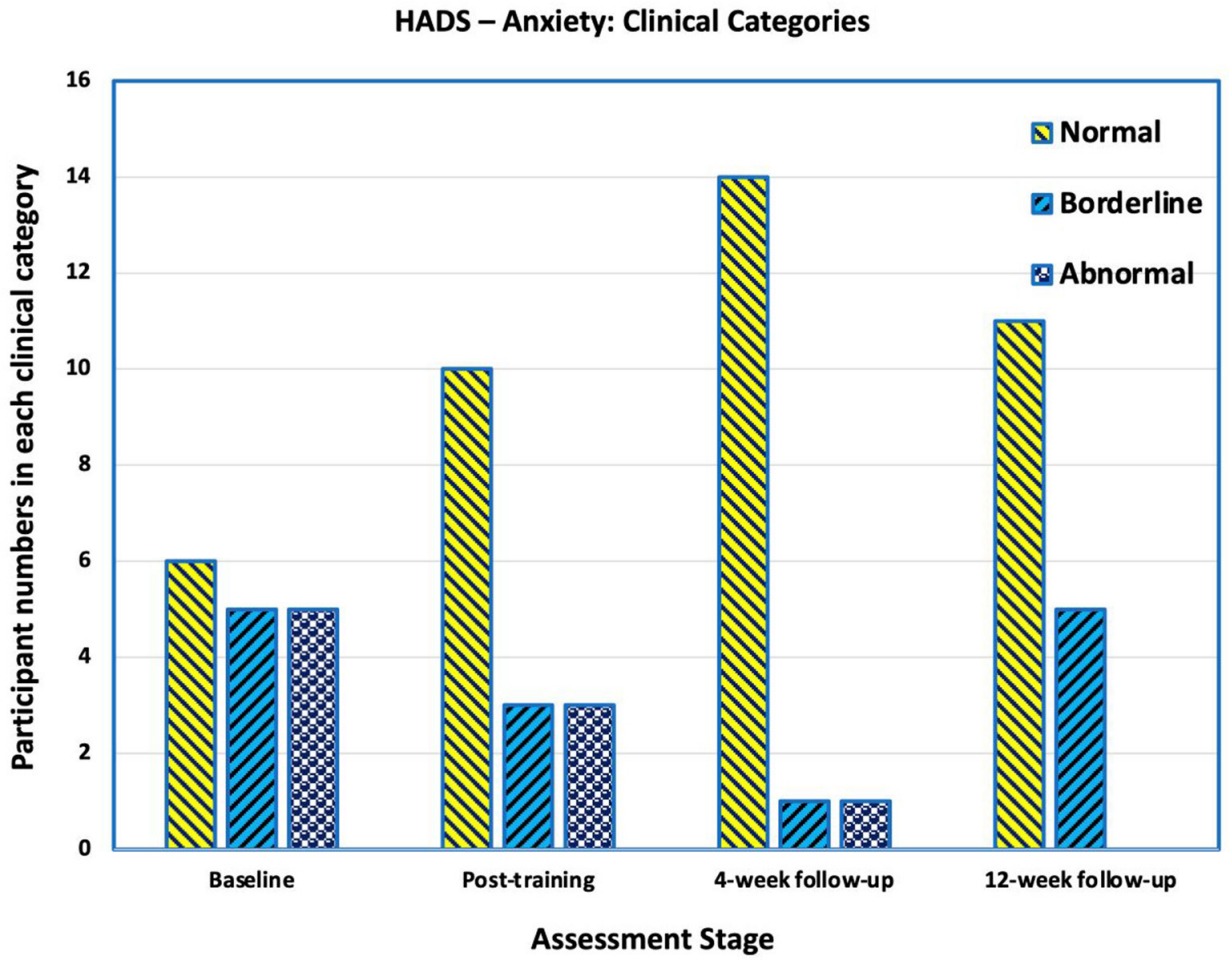
Number of participants in each category in Hospital Anxiety and Depression Scale (HADS) for Anxiety. Scores of >10 are “abnormal” and indicative of clinical anxiety, scores between 8 and 10 are categorised as borderline, and scores below 8 are categorised as normal. At baseline 6 participants had abnormal scores indicating clinical anxiety. At the 4-week follow-up, two had an abnormal score. By the final follow-up, four participants' scores changed to borderline, three deteriorating and one improving.

Prior to training six participants' Hospital Anxiety and Depression Scale (HADS)-anxiety scores were >10 indicating clinical anxiety, four were borderline cases (scores 8–10) and six were normal i.e., a score of <8 (Figure 7). At the 4-week follow-up, 13 participants had normal scores, one was borderline, and two had an abnormal score. By the final follow-up, four participants' scores changed to borderline, three deteriorating and one improving.

The mean HADS-anxiety score at the end of training (mean 6.6, SD 3.3) approached a statically significance difference compared with prior to the intervention (mean 9.3, SD 4.5; *p* = 0.178); however, there was statistically significant improvement at the later follow-up times [4-weeks and 12-weeks means of 5.6 (SD 3.7) and 5.33 (SD 3.1) respectively], compared with pre-intervention (*p* = 0.004 and *p* = 0.01 respectively).

#### Depression

**Figure 8:**
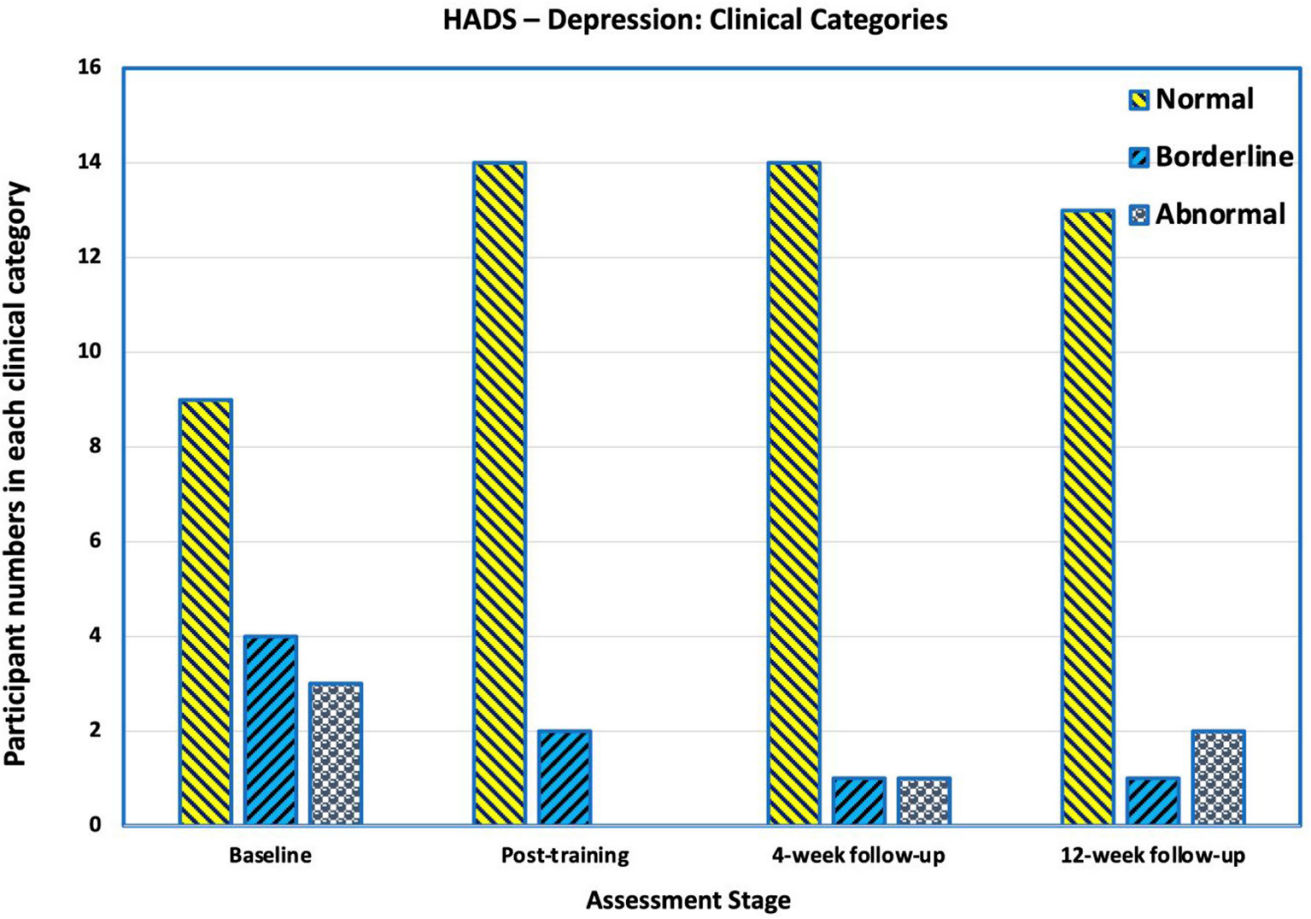
Number of participants in each category in Hospital Anxiety and Depression Scale (HADS) for Depression. Scores of >10 are “abnormal” and indicative of clinical depression, scores between 8 and 10 are categorised as borderline, and scores below 8 are categorised as normal. Before training, three participants had scores indicating clinical depression. After training, none had scores indicating clinical depression. There was a slight deterioration at 4- and 12-weeks' follow-up.

Before training, three participants had scores indicating clinical depression (>10), three were borderline (scores 8–10) and 10 normal (scores < 8). After training, 14 were normal, two were borderline and none had scores indicating clinical depression. There was a slight deterioration at 4–and 12-weeks**'** follow-up (Figure 8).

HADS-depression scores statistically significantly improved from pre-intervention levels (mean 6.8, SD 4.8) to the post-training assessment (mean 3.6, SD 2.7 *p* = 0.049). The mean HADS-depression scores at 4-weeks and 12-weeks post-training showed improvement compared to the baseline, but were not statistically significant (*p* = 0.07 and *p* = 0.137 respectively).

### EQ-5D-5L

#### EQ-5D VAS

**Figure 9:**
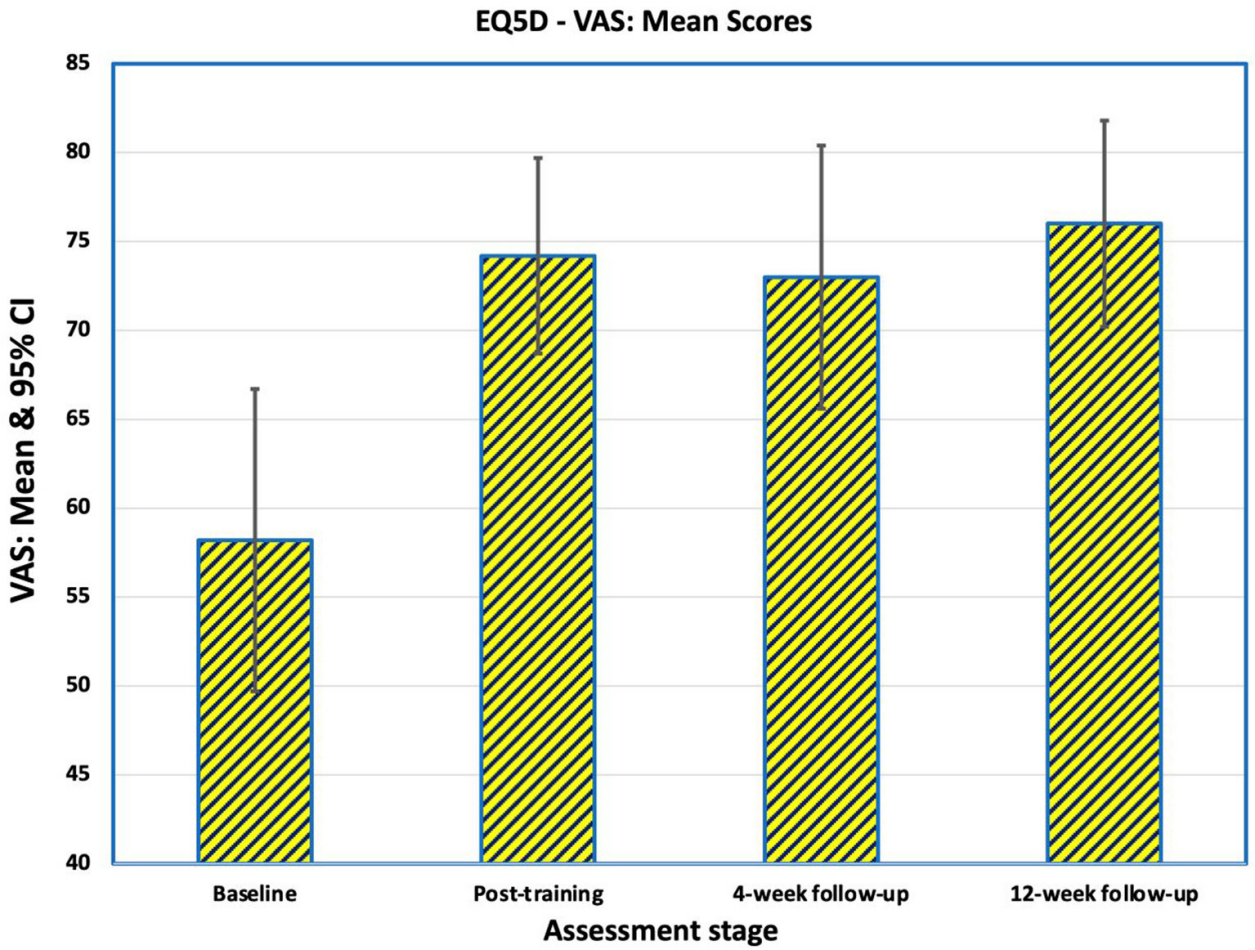
Mean scores of the 16 participants on the EQ-5D VAS captures the respondent's overall assessment of their health on a scale from 0 (worst health imaginable) to 100 (best health imaginable). After training 11 participants rated themselves as better, two were the same and three were worse compared to baseline. At the 4-weeks follow-up, 12 were better, three were the same and one was worse. At the end of the study, 12 were better, four were not improved from baseline and none were worse.

The EQ-5D VAS captures the respondent's overall assessment of their health on a scale from 0 (worst health imaginable) to 100 (best health imaginable). Mean EQ-5D VAS before training was 58.2 (SD 17.4), rising to 74.2 (SD 11.2) at the end of training (Figure 9). There was little further change at 4-weeks (mean 73.0, SD 15.2) and 12-weeks post-intervention (mean 75.9, SD 11.9). All three post-training means were significantly different from baseline (post-training: *p* = 0.012 at 4-weeks follow-up: *p* = 0.022 at 12-weeks follow-up: *p* = 0.004).

Considering change of QoL, after training 11 participants rated themselves as better (10-point improvement or greater), two were the same (0–10 points change) and three were worse compared to baseline. At the 4-weeks follow-up, 12 were better and four were the same as at baseline. At the end of the study, 11 were better, five were not improved from baseline and none were worse (Table 4).

**Table 4:** Categorical results for the primary and secondary outcome measures at the 12-week follow-up assessment stratified by degree of improvement in pain and number of NFB training sessions.

**PROM**	**Outcome**	**All participants (*n* = 16)**	**Change in VNS >30% (*n* = 8)**	**Change in VNS <30% (*n* = 8)**	**Training sessions 32–48 (*n* = 9)**	**Training sessions >48 (*n* = 7)**
VNS pain	Better	11	**–**	**–**	7	4
	Same	3	**–**	**–**	1	2
	Worse	2	**–**	**–**	1	1
CSI-A	Better	16	8	8	9	7
	Same	0	0	0	0	0
	Worse	0	0	0	0	0
PSQI	Better	13	6	7	7	6
	Same	1	1	0	0	1
	Worse	2	1	1	2	0
HADS-A	Better	12	4	8	7	5
	Same	3	3	0	1	2
	Worse	1	1	0	1	0
HADS-D	Better	12	5	7	6	6
	Same	1	1	0	1	0
	Worse	3	2	1	2	1
EQ5D-VAS	Better	11	4	7	6	5
	Same	5	4	1	3	2
	Worse	0	0	0	0	0

*CSI-A, Central Sensitisation Inventory Part A; EQ-5D VAS, EuroQuol 5 domain visual analogue score for quality of life; HADS-A, Hospital Anxiety and Depression Scale-anxiety; HADS-D, Hospital Anxiety and Depression Scale–depression; PROM, patient-reported outcome measure; PSQI, Pittsburgh Sleep Quality Index; VNS, visual numeric scale for pain.*

*There were no significant differences in the secondary outcomes when dichotomised above and below the threshold of clinically significant pain improvement (cut-off value 30% using the VNS) or the maximum per protocol number of training sessions (cut-off value 48 sessions). A further analysis with a cut-off at the mean number of training sessions (49.0) was very similar*.

#### EQ-5D-5L Profiles

Clustering of the EQ-5D-5L profiles (31) provides a categorical view of change after training with Levels 1 and 2 (No/Slight problems) representing low functional impact and Levels 3, 4 and 5 (Moderate/Severe/Extreme problems) representing medium/high functional impact.

Change of QoL at the final follow-up using clustered profiles showed 14 participants had improved QoL, one was the same and one had a mixed profile (better and worse individual responses within a profile).

Correlations between EQ-5D VAS and EQ-5D-5L profiles were examined at baseline and final follow-up. At baseline there was weak negative correlation (*r*= −0.14), but at 12-weeks follow-up the negative correlation was relatively strong (*r* = −0.47).

### Relationship Between Changes in Pain, Associated Symptoms, and Training Time

Participants' results were categorised as “better,” “the same” or “worse” for VNS, CSI-A, PSQI, HADS-anxiety, HADS-depression and EQ-5D VAS (Table 4). For the non-VNS patient-reported outcomes, the influence of clinically significant improvement in pain and number of training sessions was assessed per category. For VNS just the training session analysis is provided. For each outcome measure, similar numbers of participants were reported per category regardless of whether they achieved a clinically significant improvement in pain or the length of training. The small numbers in each group do not allow meaningful statistical analysis indicating that larger sample sizes are required to show whether there is either improvement with pain reduction or length of training.

### Changes in EEG

**Figure 10:**
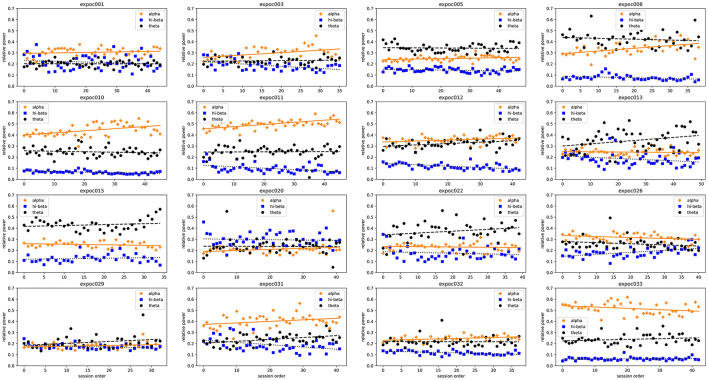
Trends in resting-state relative alpha, hi-beta and theta of each individual patient at each neurofeedback training session.

The individual per-session resting-state relative EEG power for each participant are displayed in Figure 10. This shows how resting-state alpha, hi-beta and theta changed during the NFB training period.

In summary, most patients who upregulated relative alpha or down-regulated relative hi-beta reduced their pain (VNS) and improved their CSI, PSQI, HADS, EQ5D5L depression/anxiety and VAS scores after neuromodulation. A chi-square test of independence of variables was performed to assess the correlations of EEG changes after neuromodulation with all measured pain outcomes (Supplementary Table 1). The results show a statistically significant correlation between hi-beta and HADS-Anxiety. The Chi-square tests between the rest of the variables could not reject the null hypothesis at the 95% confidence interval (*p* > 0.05), resulting in no statistically significant correlation between the two variables.

#### Relative Alpha

The comparison between pre- and post-intervention alpha activity (group means of 0.299 and 0.323, respectively) demonstrated a statistically significant modulation in relative alpha bands (*p* < 0.05) under the 95% confidence interval (Table 5).

**Table 5:** Relative alpha and hi-beta self-regulation over intervention, as shown by pre- and post-intervention activity.

**Participant**	**Number of NFB sessions**	**Resting-state relative alpha**		**Resting-state relative hi-beta**	
		**Pre-intervention**	**Post-intervention**	**Pre-intervention**	**Post-intervention**
001	45	0.259	0.328	0.289	0.186
003	36	0.257	0.283	0.222	0.175
005	48	0.230	0.239	0.149	0.148
008	39	0.300	0.348	0.073	0.07
010	46	0.410	0.443	0.076	0.061
011	46	0.453	0.534	0.159	0.061
012	43	0.339	0.365	0.136	0.089
013	50	0.246	0.232	0.222	0.164
015	35	0.242	0.233	0.116	0.108
020	42	0.205	0.269	0.334	0.282
022	40	0.210	0.229	0.242	0.165
026	41	0.332	0.284	0.167	0.237
029	33	0.18	0.209	0.189	0.164
031	42	0.347	0.389	0.236	0.173
032	38	0.228	0.26	0.129	0.114
033	43	0.548	0.529	0.057	0.07
Group mean		**0.299**	**0.323**	**0.175**	**0.142**
*p*-value		**0.013**		**0.009**	

*Pre- and post-intervention, average of first 5 sessions and last 5 sessions (pre-training baseline, eyes-opened block), respectively; p-value derived from two-tailed t-test and statistically significant under the 95% confidence interval (p < 0.05). The average relative alpha/hi-beta power of all participants and the p-values of the paired t-test are highlighted in bold*.

Eleven participants showed improvements in VNS, nine of whom had increased their relative alpha activity from pre- to post-intervention. Of those nine, seven participants showed an upward trend between the session order and relative alpha of the corresponding session (*r* ≥ 0.2). This was a statistically significant upward trend in five out of the eight participants (linear least squares regression: *p* < 0.05).

Reversing the analysis, 11 of the 16 participants (69%) showed upregulated relative alpha of whom nine showed an improvement in VNS. Six of the nine had clinically significant improvements in pain (VNS improvement >30%). These results demonstrate that the majority of patients who upregulated alpha activity also reported an improvement in reported pain.

#### Relative Hi-Beta

The comparison between pre- and post-intervention hi-beta activity (group means of 0.175 and 0.142, respectively) demonstrated a statistically significant modulation in relative hi-beta bands (*p* < 0.05) under the 95% confidence interval (Table 5).

Fourteen out of 16 participants had decreased their relative hi-beta activity at the end of their training. Linear least-squares regression analysis of the session order and relative hi-beta indicated 10 participants had downregulated relative hi-beta. Of these 10, eight (80%) showed improvements in VNS. Of the eight participants who downregulated relative hi-beta and had improved VNS scores, seven showed a statistically significant negative correlation (*p* < 0.05) between the session order and downregulation of relative hi-beta.

#### Relationship Between Hi-Beta Downregulation and Anxiety and Depression

79% of participants (11 out of 14) who decreased relative hi-beta decreased their anxiety levels in HADS, 45% of which (five out of 11) were statistically significant (*p* < 0.05). 86% of participants (12 out of 14) who decreased relative hi-beta decreased their depression levels in HADS, 50% of which (five out of ten) were statistically significant (*p* < 0.05).

### Adverse Events

No serious adverse events were reported at any point during or after the intervention.

## Discussion

### Main Findings

The results of this proof-of-concept trial of home-based NFB training in adults with chronic pain, showed that the *Axon* system provided a clinically relevant and often statistically significant improvement in chronic pain and associated symptoms in a proportion of participants.

NFB training initially reduced VNS pain scores in 11 (69%) participants, and it provided a clinically significant reduction in pain intensity, of at least 30%, for 8 (50%) that persisted at least until the 12-weeks follow-up. These results were similar to those found by Vučkovic et al. (17).

Twelve participants reported improved QoL score and four were unchanged at 12-weeks' follow-up, compared to baseline. 15 participants had improved CSI scores and one remained the same. The PSQI scores decreased in 15 and increased in one after training. The participant whose PSQI score increased already had a good sleep (PSQI = 3) at baseline and continued to do so after training (PSQI = 5). Ten participants presented with clinical or borderline anxiety at baseline and nine improved after training. Six participants presented with depression at baseline and all of them improved after training. None of these outcomes were significantly different when stratified for the degree of pain improvement (>30% or not), or for the total amount of training (>48 sessions or not).

These results suggest *Axon* NFB training may have a role in improving QoL in people with chronic pain. The narrow metric of a clinically or statistically significant change in the VNS for pain, does not describe the full experience of the participants in this trial, perhaps reflecting the known complexity of the components of peoples' chronic pain experience. Although the VNS is a useful tool to measure intensity of pain, it only provides an indication of pain at that timepoint rather than considering variable levels of pain over time and the interference of pain on daily living, which is a hallmark of chronic pain.

Central sensitisation is known to negatively impact QoL, mood and sleep (1). Our results show that CSI-A scores were significantly reduced, which may indicate that neuroplastic changes had facilitated a reduction in hypersensitivity to stimuli and a decreased receptive field to evoked pain responses. This reduction was reflected in improved QoL, sleep quality and mood.

Sleep disruption and chronic pain are comorbid disorders, with a survey of individuals suffering from fibromyalgia putting non-restorative sleep before pain in order of symptom severity (10, 35). Therefore, both should be assessed when considering treatments/interventions and healthcare costs in people with chronic pain (36). Since there is evidence for the co-occurrence of sleep disturbance and chronic pain (10), treatment with neuromodulation may be a beneficial therapy for patients with both disorders, as suggested by our results.

People with complex chronic pain can be difficult to assess and treat (15), therefore clinicians must rely on how an individual describes the intensity and nature of their symptoms, which often fluctuate (37, 38). In essence, the manifestation of a person's chronic pain may be quite variable which could account for the failure of medication to be effective in many cases. Analysis of the trends for improvement in the patient reported outcomes showed that 11 of 16 (69%) participants could be considered as “responders to the treatment,” i.e., had improvements in at least 5 of the 6 outcomes assessed at 12-weeks follow-up compared with baseline. Five participants (31%) had mixed responses with 3 or 4 outcome scores better at the end of follow-up. There were no “non-responders” ie., participants who had <3 improved outcomes.

However, the results of this trial suggest it is possible that NFB training can be effective in changing several of the components of chronic pain concurrently, which is then reflected in improvement of the broad measure of QoL. Furthermore, neuromodulation of alpha activity was shown to be a true effect, as participants had significantly modulated alpha activity post-intervention compared with pre-intervention. This indicates that *Axon* NFB led to participants modulating their own alpha activity, which has been demonstrated to be a longitudinal effect over many training sessions (16, 34). It also supports the use of purposely designed low stimulus NFB games, which allowed the participant to feel relaxed but not overstimulated, indicating the stimulus was appropriate and effective. Importantly, NFB training with *Axon* was shown to be low risk, with no serious adverse events reported. These results are consistent with a meta-analysis of randomised controlled trial (RCTs) evaluating the effectiveness and safety of NFB in chronic pain patients concluded that NFB was an effective and safe therapy in alleviating pain and pain-associated symptoms in this population (16).

### Useability

A benefit from this type of home-based NFB intervention is ease-of-use, which was clearly demonstrated during this proof-of-concept trial. Participants quickly became proficient in using the headset and tablet for NFB training.

Globally the economic impact of chronic pain is enormous both directly e.g., healthcare treatments, and indirectly through work absenteeism, loss of productivity, and the need for carers (39, 40). Development of portable NFB devices has led to a wider applicability of the technology (17) and low-cost interventions that remotely allow an individual to self-manage their pain may help to reduce societal costs associated with managing chronic pain. Our results suggest *Axon* may be able to fulfil this role in a proportion of people with chronic pain.

### COVID-19

The advantages for patients of being able to self-manage chronic pain away from a clinical setting have been highlighted during the COVID-19 pandemic. Clinic visits and access to physical and psychological therapies were limited because of lockdowns and increased waiting times. This led to undertreatment and symptom deterioration, especially pain intensity and symptoms of psychological distress, for some chronic pain patients (7, 8, 41). Participants in the trial reported that taking part in the NFB intervention during COVID-19 gave them an increased sense of empowerment during a time when other interventions or treatments were more difficult to access. Also, because participants could not access their regular medical, physiotherapy, and other rehabilitation treatments in the usual way it was easier for them to recognise that their improvements were likely to be due to NFB training.

As a result of COVID-19 lockdowns, chronic pain symptoms generally increased among sufferers (9), whereas most of our participants experienced a decrease of symptoms despite the same level of national confinement. The advantage of having a portable therapy device with fully remote connectivity has been serendipitously established during a time of global distress. As the impact of COVID-19 continues in many countries, the need for home-based non-pharmacological interventions that enable patients to self-manage pain remotely may come to be considered a cornerstone for future pain management (7). Portable NFB devices are specifically designed for such homecare interventions and allow for subjective and objective online patient monitoring through both validated patient outcome measures and analysis of brain activity.

### Limitations

As this was a proof-of-concept trial, the main limitation was the low number of participants. However, as the objective of this study was to establish the safety, efficacy and feasibility of using the *Axon* system at home to treat the primary and secondary symptoms of chronic pain, the sample size was not required to be powered for a RCT. Therefore, future larger studies will be blinded and employ a sham-control condition. This will help ensure that the volunteers are representative of the wider population who experience chronic pain and counteract any unintentional bias as much as possible. For example, involvement in research of any kind may provide an unintentional performance bias such as the “Hawthorne” effect (42), or an “intention to treat” effect which may be amplified due to participants' knowledge of the allocated intervention (43). This will also address the unproven premise that the beneficial effects attributed to NFB are due to the placebo phenomenon (44). The larger, ongoing studies also address the limited nature of the VNS tool, which captures pain at a single moment in time. These studies will include more fine-grained pain measurements that account for the fluctuations in frequency and severity associated with chronic pain.

The limited population (*n* = 16) and lack of control group in this proof-of-concept study was especially important for the interpretation of data to detect meaningful clinical changes. However, the results have been sufficiently positive to provide confidence to move forward with larger studies, especially as this study demonstrated that the intervention allowed participants to upregulate their own relative alpha activity, addressing suppressed alpha oscillations, which are known to be associated with the chronic pain disease state (16).

## Conclusions

Chronic pain is a complex multifactorial condition that is often difficult to manage. This proof-of-concept trial showed relief of primary and secondary symptoms of chronic pain using the *Axon* home-based EEG neurofeedback system in most participants. As a home-based, non-invasive device it proved to be easy-to-use, and it was not associated with any adverse events. The results were encouraging enough to justify further investigation of the technology in larger studies in more diverse patient groups. The use of such a device may prove a considerable benefit for clinicians seeking to assist chronic pain patients to self-administer treatment remotely.

## Data Availability Statement

The raw data supporting the conclusions of this article will be made available by the authors, without undue reservation.

## Ethics Statement

The studies involving human participants were reviewed and approved by Northwest England NHS Research Ethics Committee. The patients/participants provided their written informed consent to participate in this study.

## Author Contributions

NB, JG, and CO designed the study, collected, analysed and interpreted the data, and wrote the manuscript. FA and KK collected, analysed and interpreted the data, and reviewed the paper. All authors contributed to the article and approved the submitted version.

## Conflict of Interest

This proof-of-concept study was funded by Exsurgo Ltd. The funder had the following involvement with the study: study design, collection, analysis and interpretation of data, the writing of this article and the decision to submit it for publication. CO, KK, and FA are employed by Exsurgo Ltd. NB and JG are employed by East Midlands Spine Ltd. and PhysioFunction Ltd., respectively.

## Publisher's Note

All claims expressed in this article are solely those of the authors and do not necessarily represent those of their affiliated organizations, or those of the publisher, the editors and the reviewers. Any product that may be evaluated in this article, or claim that may be made by its manufacturer, is not guaranteed or endorsed by the publisher.

## Supplementary Material

Supplementary Table 1 Cross-tabulation of the number of patients who reported changes in each EEG band against each pain outcome measure. Chi-square test results comparing correlation of alpha, hi-beta and theta changes with all outcome measures. For all outcome mesures except EQ5D5L VAS, decrease of the score indicates an improvement from pre-to-post intervention. For EQ5D5L VAS, increase of the outcome measure represents improvement in quality of life from pre-to-post intervention.
